# Synthesis, Structure-Activity Relationships (SAR) and *in Silico* Studies of Coumarin Derivatives with Antifungal Activity

**DOI:** 10.3390/ijms14011293

**Published:** 2013-01-10

**Authors:** Rodrigo S. A. de Araújo, Felipe Q. S. Guerra, Edeltrudes de O. Lima, Carlos A. de Simone, Josean F. Tavares, Luciana Scotti, Marcus T. Scotti, Thiago M. de Aquino, Ricardo O. de Moura, Francisco J. B. Mendonça, José M. Barbosa-Filho

**Affiliations:** 1 Pharmaceutical Science Department, Federal University of Paraíba, João Pessoa 58051-900, PB, Brazil; E-Mails: rodrigosantos@ltf.ufpb.br (R.S.A.A.); felipeqsguerra@gmail.com (F.Q.S.G.); edelolima@yahoo.com.br (E.O.L.); josean@ltf.ufpb.br (J.F.T.); 2 Laboratory of Synthesis and Drug Delivery, Biological Science Department, State University of Paraíba, João Pessoa 58020-540, PB, Brazil; E-Mails: luciana.scotti@gmail.com (L.S.); ricardo.olimpiodemoura@gmail.com (R.O.M.); 3 Department of Physics and Informatics, Physical Institute of São Carlos, University of São Paulo, São Carlos 13560-970, SP, Brazil; E-Mail: casimone@ifsc.usp.br; 4 Engineering and Environment Department, Federal University of Paraíba, Campus IV, Rio Tinto 58297-000, PB, Brazil; E-Mail: mtscotti@gmail.com; 5 Institute of Chemistry and Biotechnology, Federal University of Alagoas, Maceió 57072-970, AL, Brazil; E-Mail: thiago.wanick@gmail.com

**Keywords:** coumarin derivatives, antifungal activity, *Aspergillus* sp., structure-activity relationships (SAR), molecular modeling, principal component analysis (PCA), partial least squares regression (PLS), density functional theory (DFT)

## Abstract

The increased incidence of opportunistic fungal infections, associated with greater resistance to the antifungal drugs currently in use has highlighted the need for new solutions. In this study twenty four coumarin derivatives were screened *in vitro* for antifungal activity against strains of *Aspergillus*. Some of the compounds exhibited significant antifungal activity with MICs values ranging between 16 and 32 μg/mL. The structure-activity relationships (SAR) study demonstrated that *O*-substitutions are essential for antifungal activity. It also showed that the presence of a short aliphatic chain and/or electron withdrawing groups (NO_2_ and/or acetate) favor activity. These findings were confirmed using density functional theory (DFT), when calculating the LUMO density. In Principal Component Analysis (PCA), two significant principal components (PCs) explained more than 60% of the total variance. The best Partial Least Squares Regression (PLS) model showed an *r*^2^ of 0.86 and *q*^2^_cv_ of 0.64 corroborating the SAR observations as well as demonstrating a greater probe *N1* interaction for active compounds. Descriptors generated by TIP correlogram demonstrated the importance of the molecular shape for antifungal activity.

## 1. Introduction

The increased incidence of fungal infections, especially dangerous hospital-acquired infections and infections in immunocompromised patients has highlighted the need for new antifungal treatments. Drug-resistant fungal isolates have been reported for all known classes of antifungal drugs. As a result, mortality, morbidity, and the associated cost of medical care for fungal infections are all steadily rising. In addition, because many of the currently available drugs are toxic and have other drawbacks involving spectrum, tissue distribution, central nervous system penetration, and high cost, the number of efficacious anti-mycotic drugs is limited [1]. Many of these drugs actually produce infection recurrence, for being fungistatic and not fungicidal.

In particular, Aspergillus infections have been increasing, and while the most frequent pathogen to cause aspergillosis is *Aspergillus fumigatus*, *A. terreus*, *A. flavus* and *A. niger* are becoming increasingly common [2]. *A. fumigatus* is an opportunistic pathogen which incites disease in hosts whose local or systemic immune response has been impaired, damaged, or is innately dysfunctional. After *Aspergillus fumigatus*, *A. flavus* is the second leading cause of invasive aspergillosis, and it is the most common cause of superficial infections. Common clinical syndromes associated with *A. flavus* infections include chronic granulomatous sinusitis, keratitis, cutaneous aspergillosis, wound infections, and osteomyelitis following trauma and inoculation. In addition, *A. flavus* produces aflatoxins, the most toxic natural hepatic-carcinogens ever characterized [3].

There is an urgent need for discovery and testing of new compounds with antifungal properties. Natural products are inexhaustible as a source of chemical structures and for more than a century have been used as scaffolds for the synthesis of new drugs. The coumarins, phenolic compounds which possess a benzopyranone nucleus [4] are one of the major classes of secondary metabolites, which has been highlighted in biological studies as being (anti-HIV [5], hepato-protective [6], anti-inflammatory [7], antimicrobial [8], antimitotic [9] and antitumor [10]), and in part, due to their antifungal properties [11–13] they are believed to exert a role in plant protection against herbivorous, fungal, and bacterial infections [14].

It was recently reported that coumarin derivatives may be used effectively as antifungal agents. Johann *et al*. [11] demonstrated that coumarins isolated from plant extracts exhibit activity against certain strains of *Sporothrix schenckii* (MICs of 125–250 μg/mL), and *Cryptococcus gattii* (MIC of 250 μg/mL). Daoubi *et al*. [13] showed their antifungal activity against *Botrytis cinerea* in concentrations ranging from 25 to 200 mg/L with IC_50_ values of 33.3–157.5 mg/L. Creaven *et al*. [15] demonstrated that coumarin derivatives are active against *Candida albicans* (MIC_80_ of 4.5–246.6 μM). Jurd *et al*. [16,17] studied several coumarin derivatives showing inhibitory activity against varied strains including Candida (*Candida albicans*, *C. tropicalis*, *C. chalmersi)*, *Saccharomyces cerevisiae*, *Aspergillus flavus*, *A. niger*, *A. oryzae*, *A. glaucus*, and *Penicillium chrysogenum*.

Pursuing our research in the field [18–20], we herewith describe the synthesis and *in vitro* antifungal evaluation of coumarin derivatives against *Aspergillus* species. After antifungal evaluation, the derivatives were subjected to structure-activity relationship (SAR) analysis, electronic surface analyses, molecular modelling, and chemometrics (Principal Component Analysis (PCA), and Partial Least Square Regression (PLS)), all to extract information on structure and its relation to antifungal properties [21,22].

## 2. Results and Discussion

### 2.1. Synthesis

**Scheme 1:**
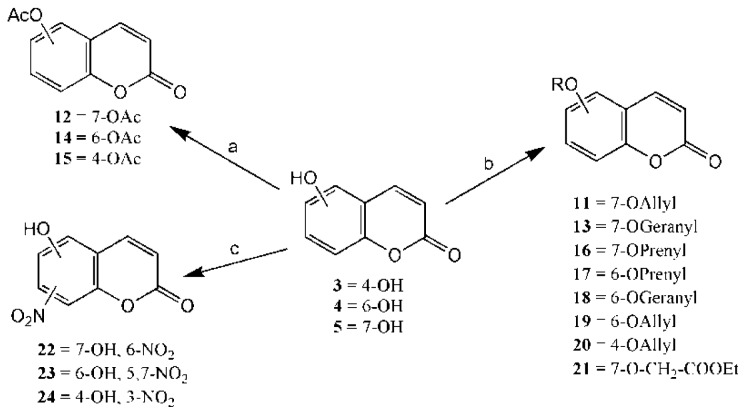
Synthesis of alkyl-, acetyl- and nitro-coumarin derivatives. Reagents and Conditions: (a) Acetic Anhydride, Pyridine, rt., ultrasound irradiation; (b) Allyl Bromide, Geranyl Bromide, Prenyl Bromide, or Ethyl Chloroacetate, K_2_CO_3_, Acetonitrile, reflux; (c) HNO_3_/AcOH, 0–5 °C for 30 min, then 90 min at rt.

As shown in Scheme 1, the coumarin derivatives (**11–24**) were synthesized by alkylation, acetylation, and nitration of commercial coumarins: 4-hydroxy- (**3**), 6-hydroxy- (**4**) and 7-hydroxy-coumarin (**5**). Alkylation reactions were done according to procedures previously described with small modifications [23,24], using different alkyl halides; (allyl bromide, geranyl bromide, prenyl bromide and ethyl chloro-acetate) which gave coumarin derivatives: **11** [25], **13** [26], **16** [26], **17** [27], **18** [28], **19** [29], **20** [30] and **21** [31] in satisfactory yields (45.5%–98%, except **18**). Acetylation was carried out under ultrasonic irradiation (which increases the rate, speed and yield of chemical reactions, by liquids emulsification [32]), using an acetic anhydride and pyridine mixture afforded derivatives **15** [33], **14** [17], and **12** [34], in good yields (77%–90%). Nitro-coumarins **22** [35], **23**, and **24** [36] were synthesized by standard nitration procedures, using a mixture of nitric and acetic acids in an ice bath [37].

The chemical structures of **11**–**22** and **24** were confirmed by comparing their physical and spectral data with the respective literatures. The purity of all synthesized compounds were >98% determined by HPLC.

Structural confirmation of **23** was based on its NMR, mass spectra and elemental analysis. In the ^1^H NMR we observed only three signals related to three hydrogens which indicated a probable bi-nitration. There were two doublets coupled together (*J* = 10.0 Hz) at 6.75 and 7.77 ppm, which were attributed to H-3 and H-4, and one singlet at 8.23 ppm (related to H-8). These signals provide good evidence that a bi-nitration occurred on the aromatic ring at the C-5 and C-7 positions, both were ortho-positioned with respect to the 6-hydroxyl group, as also observed in previous works [38]. The unequivocal determination of the carbons was done by interpretation of their ^13^C NMR, ^13^C–^1^H HMQC and ^13^C–^1^H HMBC spectra.

In the ^13^C–^1^H HMQC was possible to determine unambiguously the three primary carbons at 116.3 (C-8), 124.0 (C-3) and 136.7 ppm (C-4). Signals from quaternary carbons were determined by analysis of the correlations observed in the ^13^C–^1^H HMBC, which are described in Table 1.

The mass spectrum also confirmed the proposed structure, where it is observed as the base peak, the fragment [M-1]^+•^ at 250.9, characteristic for phenolic compounds.

### 2.2. *In Vitro* Antifungal Evaluation and SAR Study

From Table 2, the *in vitro* antifungal activity of the commercial (**1**–**10**) and synthesized coumarins (**11**–**24**) was investigated using two *Aspergillus* species, including four *Aspergillus fumigatus* strains (ATCC 16913, ATCC 40640, ATCC 46913, and IPP 210) and four *Aspergillus flavus* strains (ATCC 16013, LM-247, LM-210, and LM-26). The chemical structure, and Minimum Inhibitory Concentration (MIC) values, expressed in micrograms per milliliter compared with amphotericin B (AmpB) are presented.

The data presented in Table 2 show that commercial coumarins (**1**–**10**) are generally ineffective against the tested strains (MIC ≥ 1024 μg/mL), with the exception of coumarin **2** which presented moderately strong activity (MIC = 64 μg/mL for *A. fumigatus*, and 128 μg/mL for *A. flavus*). This difference in sensitivity between the two species was also observed for derivative **16** (MIC = 128 μg/mL for *A. fumigatus*, and 1024 μg/mL for *A. flavus*), and for the reference drug Amphotericin B (AmpB) with an MIC of 2 μg/mL for *A. fumigatus*, and between 2 and 512 μg/mL for *A. flavus*).

Despite the apparent similar sensitivity profile, it is observed that for one sixth of compounds (**7**, **11**, **12** and **20**) the *Aspergillus flavus* strain LM-247 proved to be more resistant than the other *A. flavus* strains, with MIC values of 2 to 32 times higher.

Most derivatives did not compare well to the antifungal activity of AmpB, however nine compounds showed MIC values that were either lower (**2**, **11**, **12**, **14**, **15**, **20**, **22**), or equal (**21**, **23**) to the reference drug (AmpB) for the strain LM-26 which is AmpB-resistant).

**Figure 1:**
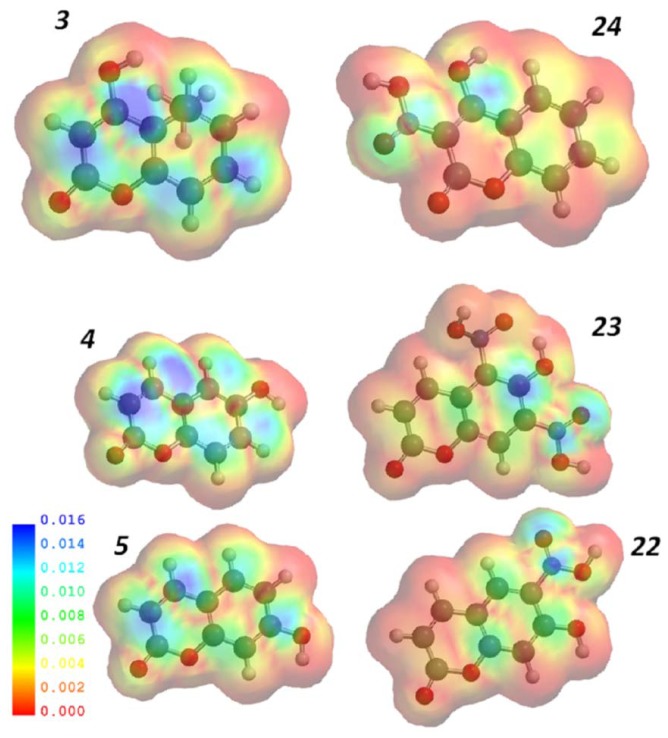
Representation of LUMO density surfaces for active (**22**–**24**) and inactive (**3**–**5**) compounds. Red regions represent lower electronic concentrations.

The introduction of electron withdrawing groups to the coumarin skeleton appears to contribute positively to the fungicidal activity, as seen with all the nitro-derivatives evaluated. Nitration of the inactive compounds (**3**, **4** and **5**) resulted respectively in derivatives: **24** (MIC = 1024 μg/mL), **23** (MIC = 512 μg/mL), and **22** the most active compound (MIC = 16 μg/mL) each derivative being (respectively) 2, 4, and 128 times more active than its precursor. DFT confirmed these findings. In the LUMO density surfaces we highlight the differences between active (nitro-coumarins) and inactive compounds. The active compounds have stronger red regions (lower electronic concentration). Although the electrostatic potential map also represents the electronic concentration, LUMO density better demonstrates the ability of one molecule to receive electrons (Figure 1).

The positive influence of electron withdrawing groups can also be observed for the three series (4-, 6- and 7-hydroxycoumarins) of synthesized compounds. In every case it was observed that the compounds resulting from acetylation (respectively **15**, **14**, and **12**), are the most active from each series. Compound **15** was as active as compound **22** (the most active compound). The hypothesis that electron withdrawing groups favor antifungal activity corroborates the observations found with PLS analysis (Section 2.3.), where it was found that the interaction of compounds with the probe *N1*, increases with antifungal activity.

Analyzing the other replacements performed (introduction of geranyl, prenyl and allyl groups), we observed differing patterns of activity for the 6-hydroxy- and 7-hydroxy-coumarin derivatives. With the 6-hydroxy-coumarin derivatives, substitutions did not result in increased fungicidal activity, all compounds showed to be inactive (**17**, **18** and **19**). This was also observed in previous studies [17]. However for the 7-hydroxy-coumarin series, there is a clear relationship between the size of the introduced group and the fungicidal activity. Reduction of the alkenyl side-chain length increases the activity proportionately, so that the compound having the largest radical (geranyl) is inactive (**13** MIC ≥ 2048 μg/mL), the compound with an intermediate side-chain (prenyl) showed strong to moderate activity (**16** MIC = 1024 μg/mL for *A. fumigatus* and 128 μg/mL for *A. flavus*), and the compound with the smallest aliphatic chain displayed the best activity profile (**11** MIC = 64 μg/mL). These results are in agreement with studies observed by Jurd *et al.* [16] who observed that the antifungal activity of umbelliferone (7-hydroxy-coumarin) may be increased by *O*-alkylation, and *O*-acylation with shorter alkyl and acyl groups.

### 2.3. Computational Studies

For performing chemometric analysis (Principal Component Analysis (PCA)), and Partial Least Square Regression (PLS) the MIC values for *Aspergillus fumigatus* ATCC-16913 were used [21,39,40]. Inhibitory activity data of the investigated compounds determined as micrograms per milliliter were converted to the negative logarithms of molar MICs (log1/*c*_MIC_) (Table 2) which was used as a dependent variables set in this study. Principal component analysis (PCA) and partial least squares (PLS) are chemometric tools for extracting and rationalizing the information from any multivariate description of a biological system. Complexity reduction and data simplification are two of the most important features of such tools. These chemometric tools were developed in the Pentacle software [41]. The Pentacle software is a computational tool for computing alignment-free molecular descriptors, also called GRid-INdependent descriptors or GRIND. The software is based on Molecular Interaction Fields, describe the ability of the molecules to interact with other molecules and do not require to superimpose the compounds. GRIND descriptors are highly relevant for describing biological properties of compounds [42] (see item 3.4. Molecular modeling and electronic surfaces).

#### Principal Component Analysis (PCA) and Partial Lest Squares Regression (PLS)

The PCA is a technique used to reduce the principal matrix information, splitting into two smaller matrices called loading and score: the matrix loading (P) contains information about the variables and is composed of vectors (principal components, PCs) which are obtained from the original variables; the matrix score (T) contains information about the objects. Each object is described in terms of the projections from the PC instead of the original variables.

Preliminary exploratory analysis was developed using PCA and considering 405 independent variables or descriptors. Two significant principal components (PCs) explain more than 60% of the total variance (Table 3).

**Figure 2:**
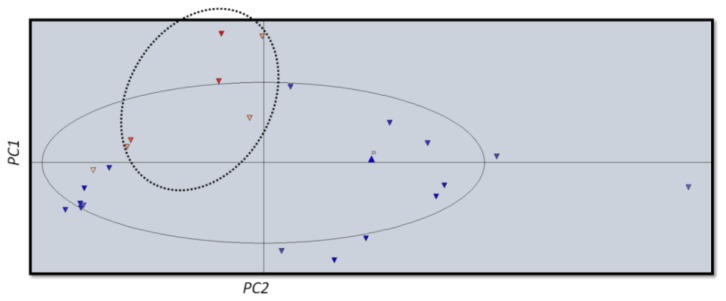
Scores plot from PCA. Red triangle represents less active and blue triangle represents more active compounds.

Score and loading plots are interconnected until any descriptor change in the loading plot is reflected by changes in the position of compounds in the score plot [43]. The score plot exhibits satisfactory discrimination between more potent (blue) and less potent (red) compounds. The less active compounds are concentrated in the upper left quadrand (Figure 2).

**Figure 3:**
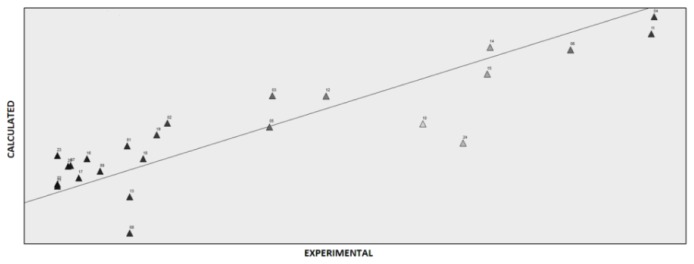
Best fit model obtained in PLS.

The best model from PLS was obtained with three LVs and 72 descriptors selected from 405. The variable selection via fractional factorial design (FFD), which evaluates the effect on the model standard derivation of error of prediction (SDEP) of every single variable and variable combination. The GRIND descriptors condensed represent a small number of principal properties (GRIND-PP), requiring a biologically relevant description of the molecular similarity. Two significant latent variables emerged from PLS model and *leave-one-out* [44]. The best model showed an *r*^2^ of 0.86, and a *q*^2^_cv_ of 0.64. Figure 3 represent the score plot obtained with PLS.

**Figure 4:**
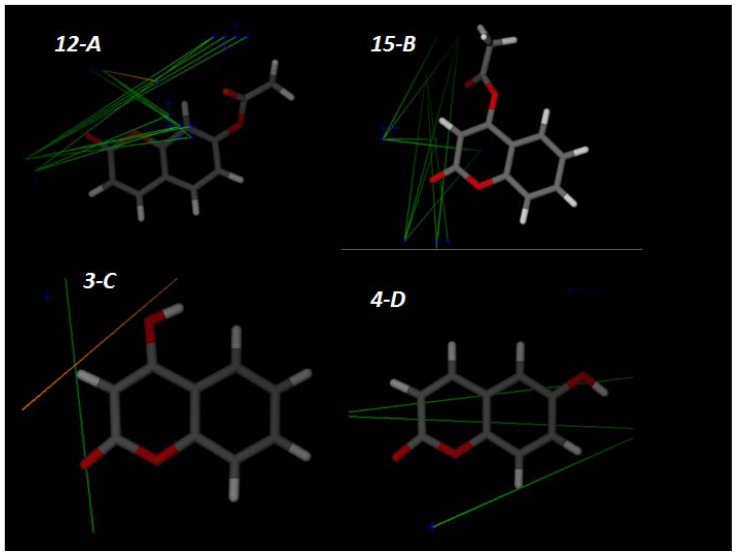
Examples of important structural features for antifungal activity, highlighted by the PLS analysis: (**A**) and (**B**) **12** and **15**, respectively—active compounds; (**C**) and (**D**) **3** and **4**—inactive compounds, respectively. *N1*–*TIP* interactions (orange), *N1*–*N1* (green) interactions.

The principal descriptors highlighted in the PLS model were: *350* (5.6–6 A), *531* (10.8–11.2 A), *527* (7.2–7.6 A), and *201* (13.2–13.6 A) were generated through interactions with the probe *TIP* (shape), the molecular contour also appears to be important. The molecular descriptors obtained can be observed in graphical diagrams called “correlograms”. We observed the largest number of interactions of active compounds with the probe *N1*. This may indicate that coumarins devoid of electrons have greater attraction with electronegative atom of this probe. Otherwise occurs in inactive compounds. The Figure 4 shows the active compounds **12** and **15** and their interactions with *N1*. Comparatively, we can see the inactive compounds (**3** and **4**). Inactive interactions are smaller than active ones, corroborating the fact that electron withdrawing groups increases the antifungal activity.

## 3. Material and Methods

### 3.1. General Methods

All synthetic coumarins (1,2-Benzopyrone **1**; 3-Hydroxycoumarin **2**; 4-Hydroxycoumarin **3**; 6-Hydroxycoumarin **4**; 7-Hydroxycoumarin **5**; 6,7-Dihydroxycoumarin **6**; Coumarin-3-carboxylic acid **7**; 3,3′-Methylene-bis-(4-hydroxycoumarin) **8**; 6-Methoxy-7-hydroxycoumarin **9** and 7,8-Dihydroxy-6-methoxycoumarin **10**), reagents and solvents were purchase from Sigma-Aldrich (Seelze, Germany), and used without further purification. All reactions were monitored by thin-layer chromatography (TLC) on pre-coated silica gel GF_254_ plates (Fluka, St. Gallen, Switzerland). Melting points were determined on a Fisaton 430 apparatus (Fisaton, São Paulo, Brazil) using open capillaries, and the reports values are uncorrected. Acetylation reactions were performed with an ultrasound USC-1400A (40 kHz, Unique, Indaiatuba, Brazil). Infrared (IR) spectra were recorded using potassium bromide pellets on a Bruker IFS-66 IR spectrometer (Bruker, San Francisco, CA, USA), with the frequencies expressed in cm^−1^. NMR were recorded on a Varian Unity Plus 300, 400 and 500 MHz spectrometer (Varian, Palo Alto, CA, USA), using TMS as an internal standard. Peak assignment in ^13^C spectra are based on 2D HSQC and HMBC spectra. Chemical shifts were reported in ppm (δ), and coupling constants (*J*) were reported in Hertz. Signals were designated as follows: s, singlet; d, doublet and bs, broad. HRMS was recorded on a MicroTOF mass spectrometer (ESI) (Bruker). Low-resolution ESI mass spectra was recorded on a Amazon X (Bruker). Elemental analysis was performed using an EA 1110 CHNS-O elemental analyzer (CE instruments, Wigan, UK).

### 3.2. Synthesis

#### 3.2.1. General Alkylation Procedure

A mixture of commercial coumarins (**3**, **4** or **5**) (3 mmol), K_2_CO_3_ (3.9 mmol), and an alkyl halides (3.9 mmol) in anhydrous acetonitrile (20 mL) was stirred in reflux for 22–46 h, and monitored by TLC. The reaction was filtered and washed two times with ethyl acetate (10 mL). The organic phase was washed with water (50 mL), dried over Na_2_SO_4_, and then evaporated in vacuum. The residue obtained was recrystallized using Methanol/THF mixtures to give the compounds (by % yield/time reaction): **11** [25] (56.7/37 h); **13** [26] (45.5/27 h); **16** [26] (90/22 h); **17** [27] (86/22 h); **18** [28] (13.5/46 h); **19** [29] (66.7/35 h); **20** [30] (78/44 h), and **21** [31] (98/38 h) as white solids.

#### 3.2.2. General Acetylation Procedure

Coumarins (**3**, **4** and **5**) (5 mmol), Ac_2_O (50 mmol), and pyridine (5.5 mmol) were mixed in a Schlenk in an ultrasound bath, at room temperature for the appropriate time (monitored by TLC). After completion, the reaction was quenched with cold water (20 mL). The mixtures were filtered, washed three times with water (20 mL), and dried in a desiccator giving pure solid products (% yield/time reaction): **12** [34] (90/15 min); **14** [17] (76.7/15 min) and **15** [33] (86/30 min).

#### 3.2.3. General Nitration Procedure

Nitro-coumarins were synthesized by standard nitration procedures [37], using a mixture of nitric/acetic acids in an ice bath for 30 min., and then at room temperature for an additional 90 min. affording: **22** [35] (84%), **23** (70%) and **24** [36] (53.3%).

*5,7-Dinitro-6-hydroxycoumarin* (**23**): Yield 70%; mp: 155–157 °C; ^1^H NMR [500 MHz, δ (ppm), cd3od]: 6.75 (d, *J* = 10.0 Hz, 1H, H-3), 7.77 (d, *J* = 10 Hz, 1H, H-4), 8.23 (s, 1H, H-8), 10.65 (bs, OH); ^13^C NMR [100MHz, δ (ppm), cd3od]: 116.3 (C-8), 118.8 (C-4′), 124.0 (C-3), 136.7 (C-4), 138.9 (C-6), 139.6 (C-5), 143.1 (C-7), 146.3 (C-8′), 159.5 (C-2); MS (EI) *m/z* (relative intensity) 250.9 ([M-1]^+•^, 100), 220.9 (40), 190.9 (10). HRMS [ESI (*m*/*z*)] calcd for C_9_H_4_N_2_O_7_ = 252.0019, found for [M-1]^+•^ = 250.9517; Anal. Calcd for C_9_H_4_N_2_O_7_: C, 42.87; H, 1.60; N, 11.11; O, 44.42; Found: C, 42.87; H, 1.61; N, 11.14; O, 44.38.

### 3.3. Antifungal Activity

The *in vitro* antifungal activity of the studied compounds was investigated using eight *Aspergillus* strains, including four strains of *Aspergillus fumigatus* (ATCC 16913, ATCC 40640, ATCC 46913 and IPP 210), and four strains of *Aspergillus flavus* (ATCC 16013, LM-247, LM-210 and LM-26). These strains were supplied by the URM Culture Collection of the Department of Mycology, Department of Pharmaceutical Sciences of the Federal University of Paraiba, Brazil. The fungi were maintained on potato dextrose agar (PDA)—Difco^®^—at 28 °C and 4 °C until testing procedures.

Stock cultures were kept on sterile Sabouraud Dextrose Agar (SDA) slants under 7 °C (±1 °C). For preparing the inoculum of the anti-mold assays, we used 7 day-old cultures grown on sterile SDA at 25–28 °C. After the incubation period, the mold conidia were removed by adding sterile NaCl 0.85% to the growth media followed by gentle shaking for 30 s. Mold conidia were counted using a hemocytometer. The conidial suspension inoculum was adjusted using sterile NaCl 0.85%, for approximately 5 × 10^6^ conidia/mL.

MIC values were determined by the microdilution broth method using 96-wells microplates [45,46]. Conidial suspension from 7-day-old *A. flavus* culture was prepared and standardized by hemocytometer in sterile NaCl 0.85% for susceptibility testing as described previously. To a 96-well plate Sabouraud broth and coumarins were added at concentrations of 2048 to 8 μg/mL. The MIC determination was conducted with an inoculum of approximately 2.5 × 10^5^ conidia/mL microorganism in each well. The plates were incubated at 25–28 °C for 72 h. Within 72 h (and confirmed at 7 days) there was visible fungal growth. The MIC was defined as the lowest concentration of antifungal agent that completely inhibited the growth of the fungi, as detected by the unaided eye [47]. Amphotericin B was used as the reference fungicide.

### 3.4. Molecular Modeling and Electronic Surfaces

Using the program Hyperchem version 8.0 [48], the chemical structures of the compounds of interest were drawn and their geometry was optimized using MM+ force field [49]. Afterwards, we performed a new geometry optimization using the semi-empiric method RM1 (Recife Model 1) [50]. The RHF (*Restricted Hartree-Fock*) level was used. The optimised structures were subjected to conformational analyses using the random search method [22,51] with 1000 interactions, 100 cycles of optimisation, and 10 lowest minimum energy conformers. The selected dihedrals were evaluated by rotation in accordance with the standard (default) conditions of the program, in which the number of simultaneous variations was 1 to 8, and acyclic chains were submitted to rotations from 60 to 180°, while torsion rings were in the range of 30° to 120°.

The lowest energy conformers selected were saved in. sdf format with the Spartan for Windows 8.0 program [52], and then exportedto the Pentacle 1.5 program [41], PCA and PLS methodologies were carried out. The Pentacle software is a computational tool for computing alignment-free molecular descriptors, also called GRid-Independent descriptors or GRIND. The software is based on Molecular Interaction Fields and describes the ability of the molecules to interact with other molecules without requiring compound superimposition. Aims to find the underlying relationship between the structure of a molecule and its binding affinity (or other biological properties) using information extracted from molecular descriptors. The calculation of descriptors includes the hydrophobic probe (*DRY*), the hydrogen bond acceptor probe (*O*), the shape probe (*TIP*), and the hydrogen bond donor probe (*N1*) [42].

The electronic surfaces were calculated in Spartan for Windows 8.0. The compared gradient was 1.02893 ×10^−7^ (red) 0.0161204 ×10^−7^ (blue).

## 4. Conclusions

Synthesis and antifungal evaluation of twenty-four coumarin derivatives against *Aspergillus fumigatus* and *A. flavus* are described. Some derivatives showed significant antifungal activities with MICs values ranging between 16 and 32 μg/mL, including seven compounds (**2**, **11**, **12**, **14**, **15**, **20** and **22**) which were more active than the reference drug Amphotericin B for the LM-26 strain (*A. flavus*).

SAR study permitted two conclusions: *O*-substitution is essential for antifungal activity and the presence of a short aliphatic chain (geranyl<prenyl<allyl), and/or electron withdrawing groups (NO_2_ and/or acetate) favors activity.

In parallel, the compounds were submitted to the chemometric tools: PCA and PLS, using the program Pentacle. The results were satisfactory and corroborated the SAR analysis. The most active compounds showed greater interaction with the probe *N1*, which shows greater electronic concentration. These findings were also confirmed by density functional theory (DFT), calculating the LUMO density. Descriptors generated by the probe *TIP* were also highlighted, indicating that the molecular contour is indeed important for the antifungal activity, as observed in the SAR analysis.

These results suggest derivatives of coumarin as promising compounds for the development of new anti-*Aspergillus* agents.

## Figures and Tables

**Table 1:** ^13^C–^1^H HMBC correlations for **23**.

Hydrogen	Carbon	C-2	C-3	C-4	C-4′	C-5	C-6	C-7	C-8	C-8′

	(ppm)	159.5	124.0	136.7	118.8	139.6	138.9	143.1	116.3	146.3
H-3	6.75	α	linked	α	β	-	-	-	-	-
H-4	7.77	β	α	linked	α	β	-	-	-	β
H-8	8.23	-	-	-	β	-	β	α	linked	α

**Table 2:** Chemical structures and *in vitro* antifungal activity of compounds **1**–**24** against *A. fumigatus* and *A. flavus*.

		*A. fumigatus*				*A. flavus*			
			
Compound	Chemical Structures	ATCC 16913 (log1/*c*_MIC_)	IPP 210	ATCC 46913	ATCC 40640	LM 247	ATCC 16013	LM 210	LM 26
**1**	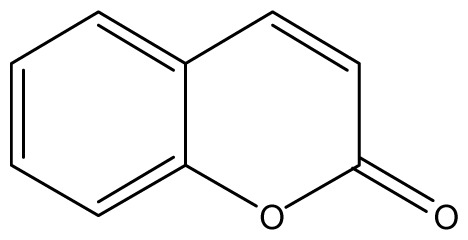	1024 (2.15)	1024	1024	1024	1024	1024	1024	1024
**2**	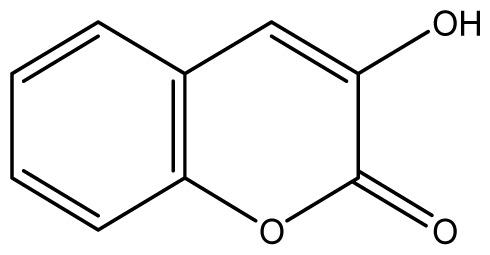	64 (3.40)	64	64	64	128	128	128	128
**3**	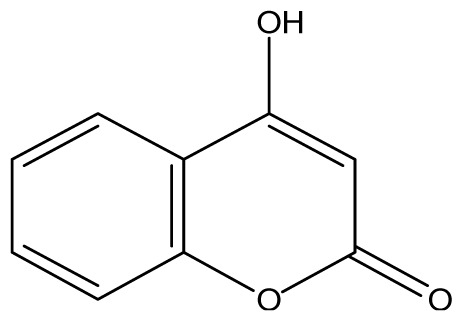	≥2048 (1.89)	≥2048	≥2048	≥2048	≥2048	≥2048	≥2048	≥2048
**4**	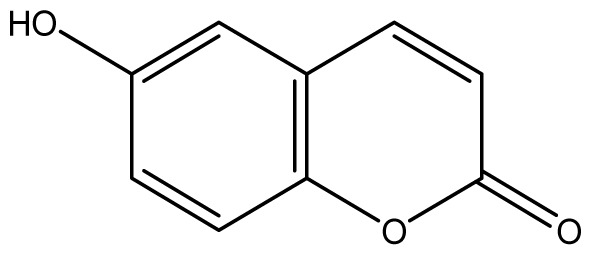	≥2048 (1.89)	≥2048	≥2048	≥2048	≥2048	≥2048	≥2048	≥2048
**5**	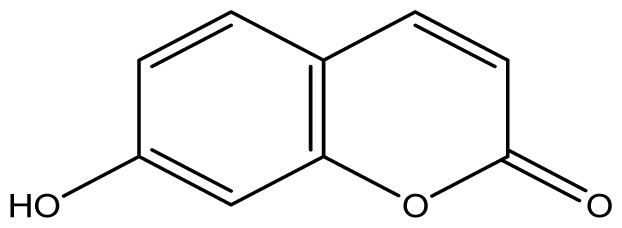	≥2048 (1.89)	≥2048	≥2048	≥2048	≥2048	≥2048	≥2048	≥2048
**6**	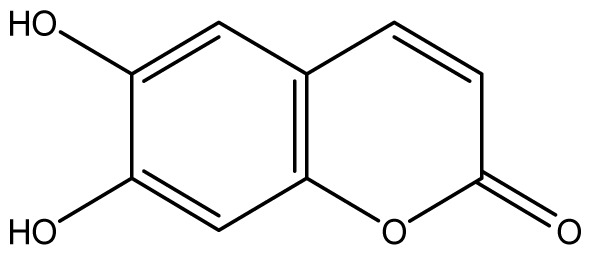	≥2048 (1.93)	≥2048	≥2048	≥2048	≥2048	≥2048	≥2048	≥2048
**7**	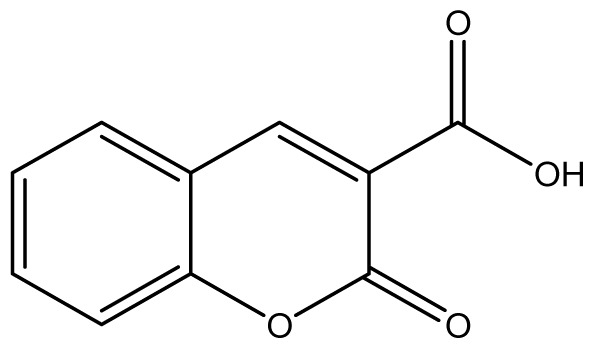	1024 (2.26)	1024	1024	1024	≥2048	1024	1024	1024
**8**	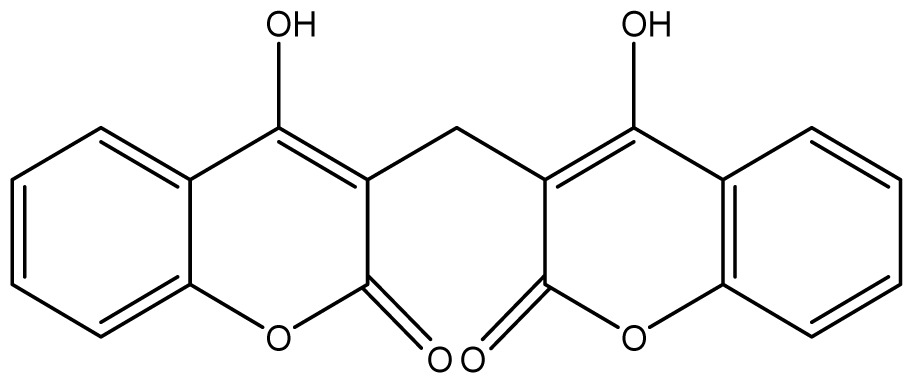	≥2048 (2.21)	≥2048	≥2048	≥2048	≥2048	≥2048	≥2048	≥2048
**9**	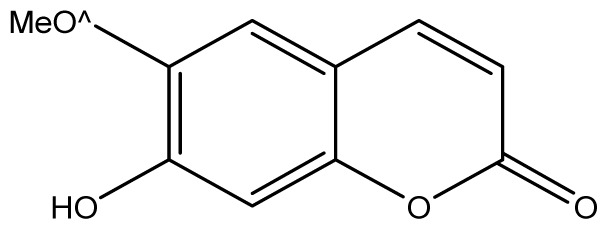	≥2048 (1.97)	≥2048	≥2048	≥2048	≥2048	≥2048	≥2048	≥2048
**10**	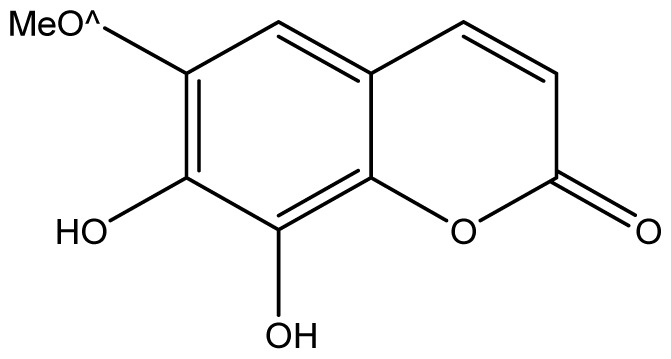	≥2048 (2.00)	≥2048	≥2048	≥2048	≥2048	≥2048	≥2048	≥2048
**11**	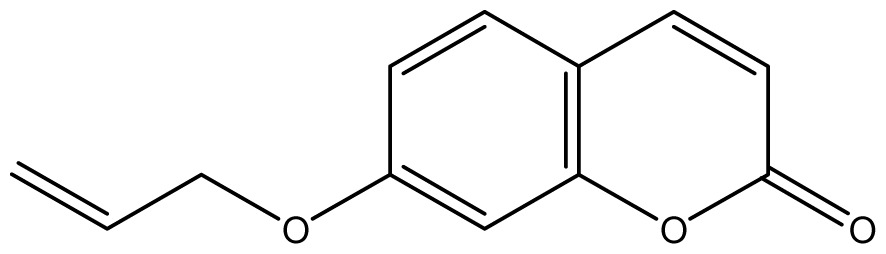	64 (3.49)	64	64	64	256	64	64	64
**12**	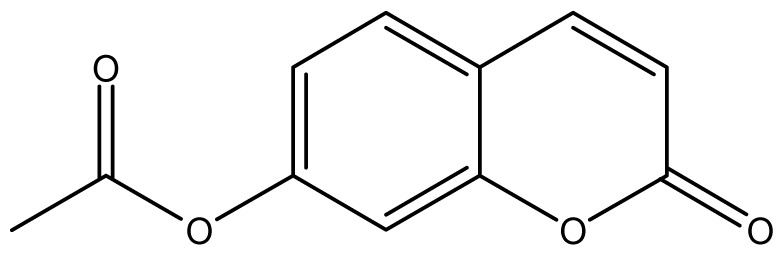	64 (3.50)	64	64	64	128	64	64	64
**13**	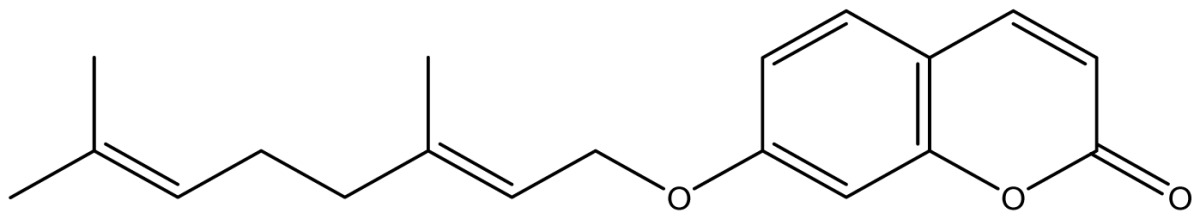	≥2048 (2.16)	≥2048	≥2048	≥2048	≥2048	≥2048	≥2048	≥2048
**14**	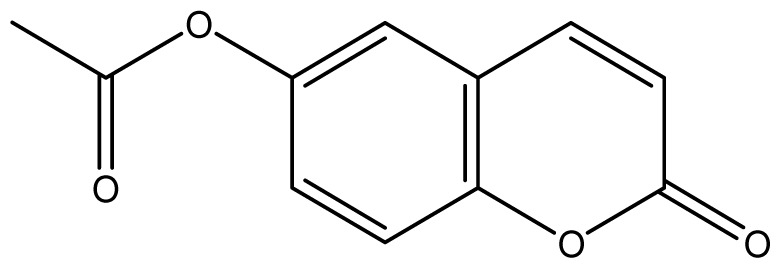	256 (2.89)	256	256	256	256	256	256	256
**15**	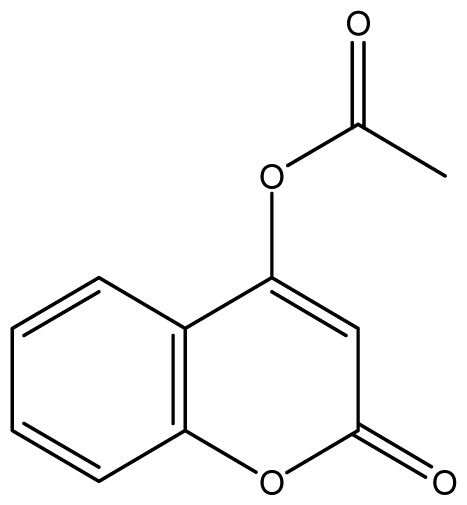	16 (4.10)	16	16	16	16	16	16	16
**16**	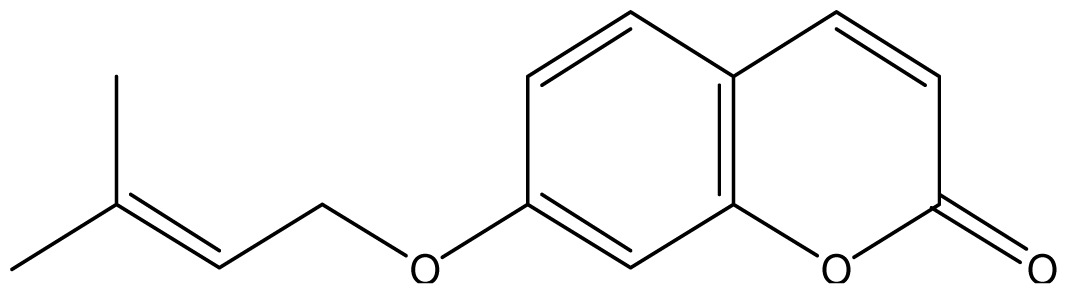	128 (3.25)	128	128	128	1024	1024	1024	1024
**17**	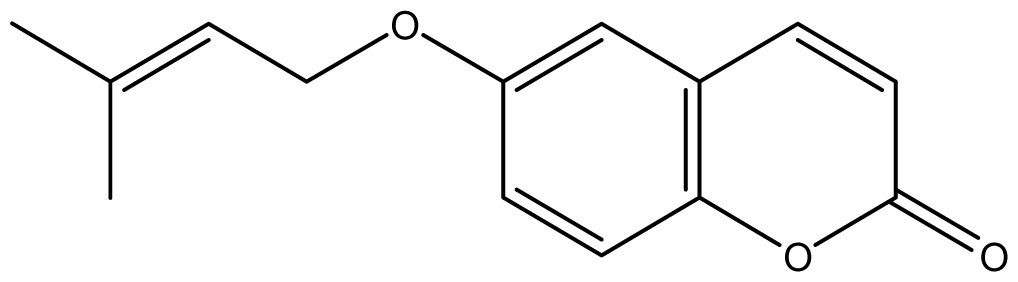	≥2048 (2.05)	≥2048	≥2048	≥2048	≥2048	≥2048	≥2048	≥2048
**18**	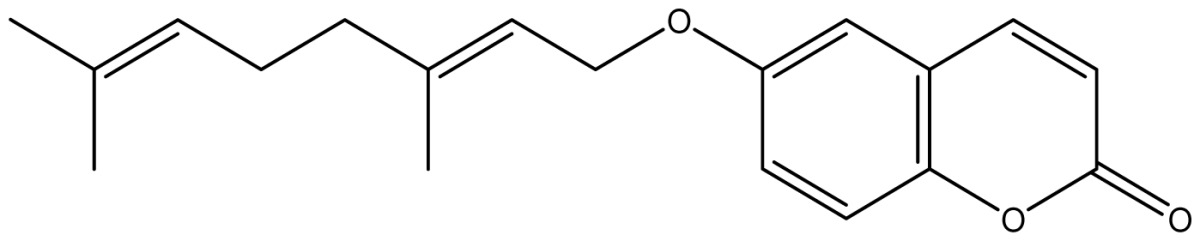	≥2048 (2.16)	≥2048	≥2048	≥2048	≥2048	≥2048	≥2048	≥2048
**19**	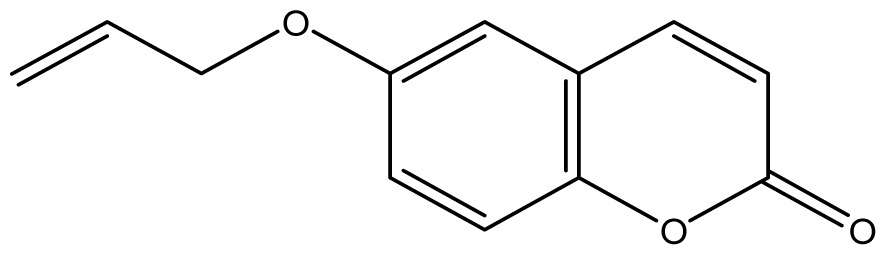	≥2048 (1.99)	≥2048	≥2048	≥2048	≥2048	≥2048	≥2048	≥2048
**20**	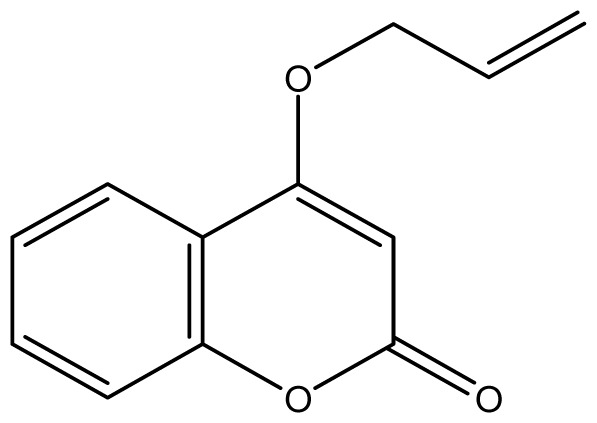	32 (3.80)	64	32	32	1024	32	32	64
**21**	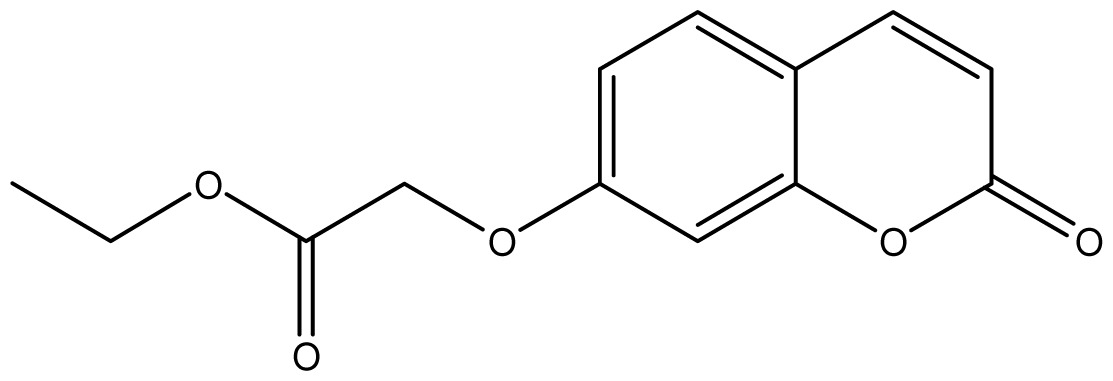	512 (2.68)	512	512	512	512	512	512	512
**22**	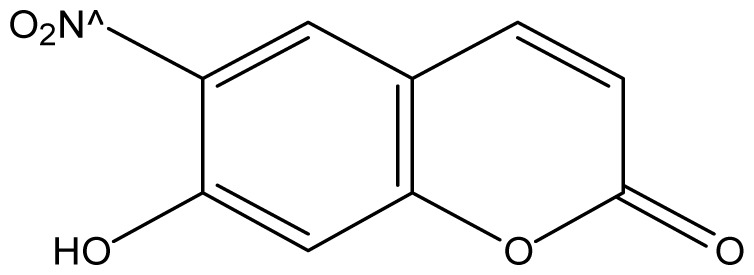	16 (4.11)	16	16	16	16	16	16	16
**23**	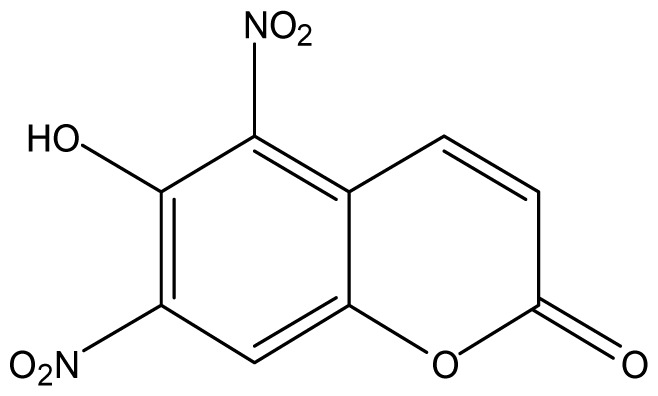	512 (2.69)	512	512	512	512	512	512	512
**24**	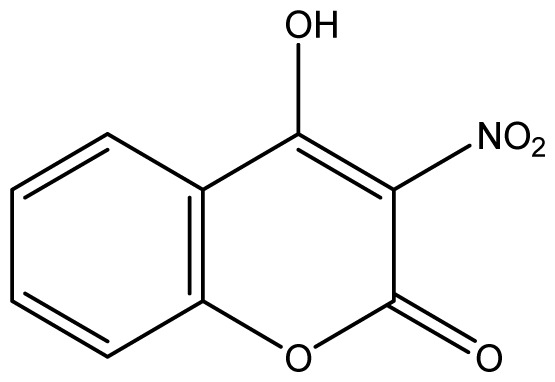	1024 (2.30)	1024	1024	1024	1024	1024	1024	1024
AmpB*		2	2	2	2	8	2	2	512

* AmpB, Amphotericin B.

**Table 3:** Explained variance using PCA.

PC	% explained variance from original data
1	50.04
2	10.62
3	7.96
4	5.80
5	3.54
